# Genetic comparison of sickle cell anaemia cohorts from Brazil and the United States reveals high levels of divergence

**DOI:** 10.1038/s41598-019-47313-2

**Published:** 2019-07-26

**Authors:** Pedro R. S. Cruz, Galina Ananina, Vera Lucia Gil-da-Silva-Lopes, Milena Simioni, Farid Menaa, Marcos A. C. Bezerra, Igor F. Domingos, Aderson S. Araújo, Renata Pellegrino, Hakon Hakonarson, Fernando F. Costa, Mônica Barbosa de Melo

**Affiliations:** 1 Laboratory of Human Genetics, Centre for Molecular Biology and Genetic Engineering (CBMEG), University of Campinas – UNICAMP, Campinas, SP Brazil; 2 Department of Medical Genetics and Genomic Medicine, Faculty of Medical Sciences, University of Campinas - UNICAMP, Campinas, SP Brazil; 3 Genetics Postgraduate Program, Federal University of Pernambuco, Recife, PE Brazil; 4 Haematology and Haemotherapy Foundation of Pernambuco – HEMOPE, Recife, PE Brazil; 5 Center for Applied Genomics, Abramson Research Center, The Children’s Hospital of Philadelphia, Philadelphia, USA; 6 Haematology and Haemotherapy Centre, University of Campinas – UNICAMP, Campinas, São Paulo Brazil

**Keywords:** Anaemia, Quantitative trait loci, Genetic markers, Genetic variation

## Abstract

Genetic analysis of admixed populations raises special concerns with regard to study design and data processing, particularly to avoid population stratification biases. The point mutation responsible for sickle cell anaemia codes for a variant hemoglobin, sickle hemoglobin or HbS, whose presence drives the pathophysiology of disease. Here we propose to explore ancestry and population structure in a genome-wide study with particular emphasis on chromosome 11 in two SCA admixed cohorts obtained from urban populations of Brazil (Pernambuco and São Paulo) and the United States (Pennsylvania). Ancestry inference showed different proportions of European, African and American backgrounds in the composition of our samples. Brazilians were more admixed, had a lower African background (43% vs. 78% on the genomic level and 44% vs. 76% on chromosome 11) and presented a signature of positive selection and Iberian introgression in the HbS region, driving a high differentiation of this locus between the two cohorts. The genetic structures of the SCA cohorts from Brazil and US differ considerably on the genome-wide, chromosome 11 and HbS mutation locus levels.

## Introduction

Sickle cell anaemia (SCA) is caused by homozygosity for a point mutation in the beta-globin gene (HBB) on chromosome 11. SCA was the first monogenic disease to be described in humans^1^ and manifestations are caused by red blood cells damaged by HbS^2^. Five RFLP-assessed haplotypes, named after the locations where they occur more frequently (Benin, Central Africa Republic or CAR, Cameroon, Senegal and Arab-Indian), are classically used to classify the HBB cluster. High fetal haemoglobin (HbF) levels are associated with the Senegal and Arab-Indian haplotypes, compared to the Benin, CAR and Cameroon haplotypes^3^. Individuals with CAR haplotypes tend to present the lowest HbF levels, while individuals with the Benin haplotype usually have intermediate HbF production levels^4^. Despite the fact that the protective effect of HbF may vary according to its distribution amongst erythrocytes, as shown by severe SCA cases carrying the Arab-Indian haploytpe^5^, these findings have motivated abundant characterization of diverse SCA populations worldwide regarding HBB haplotypes.

Some effort has been made to describe genetic diversity and structure among SCA patients^6–9^. Nonetheless, aspects regarding the effect of European ancestry^10^ and fine genetic structure on the SCA mutation locus remain elusive. Large association studies have been mostly conducted on SCA patients from the US (SUS). Other studies, such as those conducted in Brazilian SCA patients (SBR), rely frequently on findings from studies of the SUS population. The US and Brazil have the highest prevalence of new-borns with SCA on the American continent, estimated to be 4,351 and 2,978, respectively, in 2010^11^ and are divergent in demographic history regarding migration and admixture.

Here, we aim to clarify how admixed populations affected by SCA diverge from each other. Also, we aim to further describe the ancestry of SCA patients from Brazil, who have been explored relatively little compared to US patients. To achieve these goals, we propose to compare genetic structures of two populations at the genome-wide and local levels, through the analysis of a North American cohort (from the Children’s Hospital of Philadelphia-PA) and a Brazilian SCA cohort (from the Haematology-Haemotherapy Centre in Campinas-SP, HEMOCENTRO, and Haematology and Haemotherapy Foundation of Pernambuco-PE, HEMOPE), using high-density genome-wide microarrays (Genome-Wide Human SNP Array 6.0, Affymetrix Inc., CA, USA).

## Results

We evaluated the genetic structure at the genome-wide and chromosomal level by comparing SCA cohorts from the United States and Brazil, along with 19 worldwide populations from the African, European, American and Asian continents. Unaffected people (HbAA genotype) sampled from US and Brazil (AAM and BRZ, respectively), were also included (see Supplementary Table S1).

Genomic data from all populations were analysed by keeping 155,820 SNPs after quality control for a principal component analysis (PCA), depicted in Supplementary Fig. S1. As expected, BRZ genetic variation is displayed as very heterogeneous in the PCA, with individuals being dispersed between European and African populations, via a pattern demonstrated before^12,13^. We also computed Hudson’s fixation index (F_ST_) as a measure of genetic distance between populations (Supplementary Table S2) and found SBR to be closer to Europeans, relatively to SUS. PCA and F_ST_ were also concordant in depicting SCA patients from the US and Brazil as closer to each other (F_ST_ = 0.017) than to Europeans (values ranging from 0.03 to 0.088).

**Figure 1:**
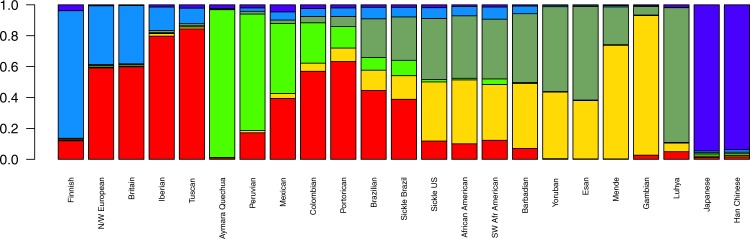
Mean ancestral components inferred by ADMIXTURE analysis. This analysis was performed using 155,820 SNPs across the genome. K = 6 had the lowest cross-validation error and thus was selected to represent ancestral components. Each bar represents a population in x-axis, while y-axis depicts mean proportional ancestry for each population (see Supplementary Table S1 for details on each population). N/W: North and West; SW: Southwest; Sickle: sickle cell anaemia.

By estimating mean ancestries for each one of the 23 populations (Fig. 1 and Table 1), we found that both European and African ancestries are predominant in the affected cohorts. Table 1 depicts mean ancestries for the SCA groups by geographical region. The South European (presumably Iberian and Italian) ancestral component estimate is more prominent in Brazilians (both affected and non-affected). Eastern African (Bantu) corresponds to a larger proportion of within-Africa ancestry in Brazilians relatively to US samples’ mean estimates. BRZ was close to SBR, except that the latter seems to have slightly more African ancestry, while SUS individuals seem to have a somewhat lower mean African component compared to AAM (Fig. 1). On a global scale, the mean ancestry of US patients is 78% African, 19% European and 1.5% Amerindian, while affected Brazilians show a mean ancestry of 43%, 45% and 10%, respectively, consistent with previous reports^8,9^. Moreover, African components are divided into 38.3% Western-Africa Mandé-related and 39.4% Eastern Bantu-related on average for the SUS, while SBR present 15.3% Mandé-related and 28% Bantu-related ancestries (Table 1).

**Table 1:** Mean (±standard deviation) ancestry proportions for sickle cell anaemia patients from the US and Brazil.

		Africa		Europe		America
		East	West	North	South	
Genomic ancestry	Sickle Cell US	39.4% (±8%)	38.3% (±8%)	7% (±4%)	12% (±6%)	1.5% (±1%)
	Sickle Cell Brazil	28% (±10%)	15.3% (±6%)	6.3% (±4%)	39% (±12%)	10% (±4%)
Chr. 11 ancestry	Sickle Cell US	76% (±6%)		18.3% (±4%)		5.7% (±4%)
	Sickle Cell Brazil	44% (±10%)		39.3% (±8%)		16.7% (±6%)

Genome-wide and chromosome 11 ancestry proportions as inferred by ADMIXTURE (at K = 6) and SABER+, respectively. Here East is considered Bantu and West is Mandinka/Mende people (Mandé group).

**Figure 2:**
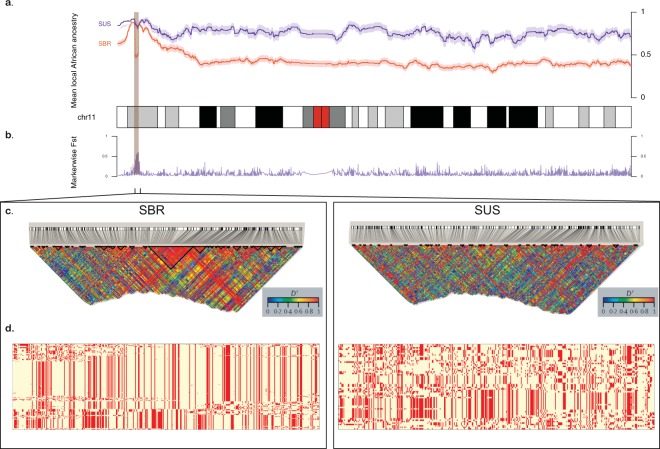
Comparison between Brazilian (SBR) and American (SUS) sickle cell anaemia patients on chromosome 11. (**a**) Diagram of the chromosome 11 (27,188 SNPs). Higher panel: x-axis represents physical position, y-axis is local mean African component inferred by SABER+; shades denote standard errors. (**b**) F_ST_ values for each marker showing high differentiation on the HBB cluster region (highlighted), also a site where SBR shows a drop in mean African ancestry. (**c)** Linkage disequilibrium in GOLD heat map generated by Haploview for SBR (left) and SUS (right) cohorts. (**d**) Phased haplotypes diagram along the highlighted area (chromosome 11:4.5–5.7 Mb) for SBR (left) and SUS (right).

We also explored ancestries on chromosome 11, where the HBB gene cluster is located (Fig. 2, Tables 1 and 2 and Supplementary Figs S2 and S3). In contrast to Brazilians, the affected American cohort typically shows more than 70% of African haplotypes along the chromosome. SBR had a more balanced constitution, showing 44% African and 39.3% European haplotypes inferred from the phased data on average (while SUS had an estimated mean of 18.3% of haplotypes of European origin), see Table 1. SCA cohorts also diverged in supposedly Native American proportions (mean 5.7% vs. 16.7% for SUS and SBR, respectively). Of note, the two populations evaluated at the chromosomal level had a similar peak, evidencing predominance of African haplotypes on 11p15.4, where the HBB region is located (Fig. 2a), except for a 1.2 Mb region where African ancestry estimates drop sharply for Brazilians.

**Table 2:** HBB locus haplotype classification for sickle cell anaemia patients from the US and Brazil.

	Sickle Cell Brazil	Sickle Cell US
CAR	73.9%	10.0%
BEN	23.4%	63.3%
CAM	0.5%	8.3%
SEN	0.0%	6.7%
AI	0.0%	0.0%
Atypical	2.2%	11.7%

Haplotypes were inferred *in silico* by haplotypeClassifier^45^. CAR: Central Africa Republic; BEN: Benin; CAM: Cameroon; SEN: Senegal; and AI: Arab-Indian.

**Figure 3:**
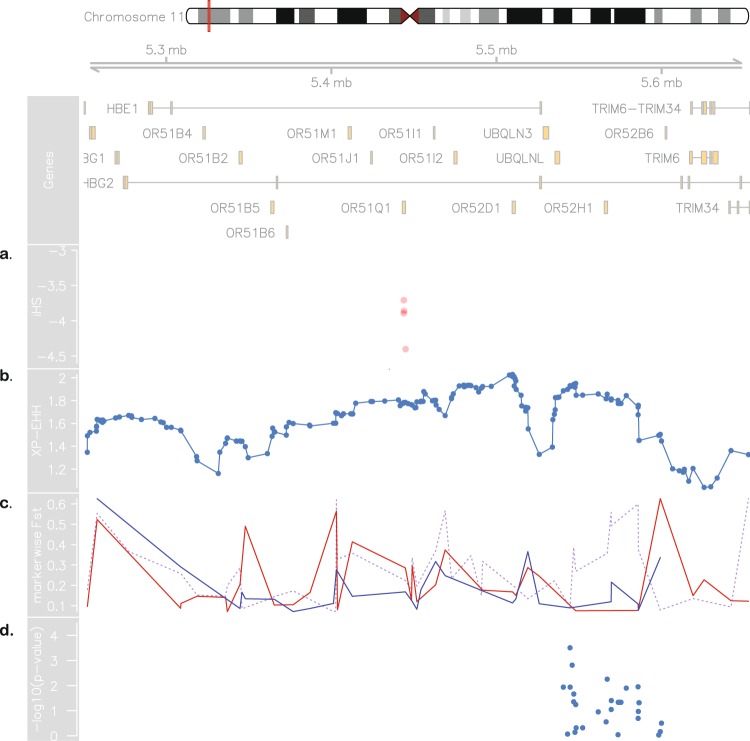
Evidence for positive selection in Brazilian sickle cell patients. At the top: chromosome 11 ideogram highlighting the region from 5.2 to 5.7 Mb, followed by genomic context. (**a)** Brazilian iHS values (values below −2 indicate positive selection). (**b)** XP-EHH between sickle cell anaemia cohorts from Brazil and US (values above 2 are considered signals of selection in one population but not in the other). (**c)** Pairwise SBR-SUS (dotted purple line), SBR-IBS (red line) and SBR-LWK (blue line) F_ST_ values. (**d)** Association between makers in the 5.45–5.59 Mb range and HbF levels in the Brazilian cohort.

We also found SUS and SBR to have highly divergent allele frequencies in a region at 11p15.4, as highlighted in Fig. 2b, measured by marker-wise Weir and Cockerham’s F_ST_ estimates. Additionally, markedly different LD and haplotype structures (Fig. 2c,d) were found in the same region. SBR subjects have a well-defined 266 kb LD block comprising the HBB cluster and a part of the locus control region (LCR), while SUS subjects have a 13 kb block upstream of the cluster and scattered regions of high LD (Fig. 2c). The conventional classification by RFLP-defined haplotypes was inferred *in silico* and conformed to expected proportions for both SCA cohorts (Table 2). We next conducted the integrated haplotype score (iHS), and found a region in which Brazilians have markers with iHS values ranging from −3.8 to −4.5 (Fig. 3a), indicating that there are alleles showing a pattern of extended haplotype homozygosity (EHH), probably a result of recent selective sweep. To test if this signal is replicated in SUS patients, we conducted a cross-population EHH calculation (XP-EHH, Fig. 3b) and found this measure to converge towards a value of 2, suggesting that a recent positive selection event took place on the SBR population near the chr11:5.4–5.5 Mb region, but not in the SUS, consistent with the abovementioned difference in F_ST_ values (also depicted for this region, dashed line in Fig. 3c).

We found local F_ST_ values on the 5.45–5.59 Mb range to be lower when comparing Iberian (IBS) and Eastern African Bantu (LWK) populations to SBR (Fig. 3c red and blue lines, respectively), suggesting that this region may present an introgression from Iberian origin. The F_ST_ values in the Bantu/Iberian comparison to SBR are still fairly high (above 0.3), consistent with a scenario of positive selection. The hypothesis of introgression is also corroborated by the European ancestry local estimates in this location (Supplementary Fig. S2). We then tested LD between the markers presenting atypical iHS values and markers around the rs334 mutation (untyped) region to evaluate if the selection signal is a product of malaria resistance and found no linkage between the two regions (Supplementary Fig. S3). We also compared SBR to BRZ and found this region to have F_ST_ values as high as 0.76 thereby ruling out the hypothesis that this signal is derived from a selective pressure that all Brazilians undergo.

Due to the implication of this region in the production of gamma globin, we performed association analysis in a SBR subset. In doing so, we found two linked SNPs to be positively associated with HbF levels after correcting for age, sex and hydroxyurea treatment and adjusting p-values for multiple testing: rs1433567, p-value = 0.0096 and rs2010794, p-value = 0.046 (see Figs 3d, S4 and Supplementary Table S3). The markers are located in the LCR region, in the olfactory receptor gene cluster upstream to HBB and have not been reported in association with HbF before. Moreover, these markers are in LD with regions comprising BCL11A biding sites described in Liu *et al*.^14^ and RFLP sites used in the HBB haplotype assignment (Supplementary Fig. S3).

## Discussion

In the present study, we compared SCA patients from the US and Brazil through the analysis of population structure at two levels, by genome-wide analysis and by further exploring the mutation-harbouring chromosome. At the genomic level, the cohorts showed substantial differences with respect to ancestry. We found the Brazilian cohort to be more admixed (Fig. 1 and Table 1) and more likely to have greater European and Amerindian ancestries, while the US sample has a more prominent African background. Brazilian ancestral proportions concur with a previous report on a sickle cell disease sample analysed on the continental ancestries level^8^.

By subdividing ancestry origins further to the subcontinental scale, the North American cohort had a pattern of within-Africa ancestry consistent with reports of genetic relatedness to Yorubans^9,15^. In a large study, Tishkoff *et al*. examined four African American populations along with 181 global populations and concluded that the former have ancestry predominantly from West-Africa (approx. 71%), followed by Europe (approx. 13%), other African regions (approx. 8%) and America (approx. 4%)^16^. They also described the African Americans to have a 45% Bantu mean ancestry and 22% non-Bantu (Mandinka ethnolinguistic group) mean ancestry, emphasizing that the diaspora encompassed a broad region in Africa, ranging from Senegambia in the west all the way to Angola, in the south^16^. Our data are consistent with these findings for both the SUS population and non-affected African descendants from the US, which are nearly identical in ancestral composition.

Brazilian affected and unaffected subjects, on the other hand, are somewhat discernible by both PCA and ADMIXTURE plots, although we assert that the non-affected sample was not controlled for skin pigmentation and was rather collected at random. More importantly, Brazilian HbAA were all collected in São Paulo, while the SCA group has also subjects from Pernambuco. Still, this differentiation is markedly small (F_ST_ = 0.001; Supplementary Table S2) and advocates for a higher admix rate in the Brazilian SCA cohort compared to the US cohort analysed. The former has two-thirds of its African heritage traced to the East-African Bantu population, and the other one-third to West-African non-Bantu populations. Although Brazilian predominance of Bantu composition is consistent with reported migration records, the SUS group shows a net contribution that is greater than what we observed for Brazilians. This might reflect the Bantu expansion, one of the major demographic movements in history of mankind, thought to have started around five thousand years ago, when Bantu-speaking people from Nigeria/Cameroon spread East and South, a migration probably prompted by agriculture^17^.

Unlike the SCA population from the US (see Solovieff *et al*.^9^), Brazilian SCA has only been briefly described in terms of genetic structure and ancestry^8^, and to the best of our knowledge, to date no subcontinental ancestry has ever been evaluated in this population. Kehdy *et al*. evaluated 6,487 subjects from the general populations of Northeast, Southeast and South Brazil, finding them to display two distinct within-Africa ancestry components: non-Bantu Western and Bantu Eastern and that the former was more prominent in Northeast Brazil, while the latter is more prominent in the South-eastern/Southern areas. Nonetheless, Bantu only accounted for an average of 36% in Southeastern people and 44% in Southern Brazilians, while we found Brazilians, irrespective of disease status, to share 65% of their African heritage traced to Bantu on average. This might be due to the different regional origins of the recruited subjects and/or other methodological and analytical aspects, although both are in agreement with historiographical data, which states that enslaved Yoruban people arrived in large numbers in the Northeast port of Salvador, whereas the Mozambican Bantu slaves disembarked largely in Rio de Janeiro ports, in South-eastern Brazil^18^. Also, Hudson’s F_ST_ on genomic markers confirms that our sample of SCA from Brazil is slightly closer to Bantu than to non-Bantu populations. SCA individuals from the US display a more even sub-continental African composition and greater proximity to the African populations evaluated here, indicating assortative mating may have had great impact on the US cohort. It is noteworthy that the F_ST_ values also show that the two affected cohorts are closer to each other than they are to European populations, and that the SBR cohort is closer also to its US counterpart than to any African population surveyed.

We found that chromosome 11 haplotype ancestries in SCA cohorts generally correspond to the genome-wide ancestry proportions we found in the previous analysis. Moreover, inferred HBB haplotypes agreed with the expected distribution: CAR prevails in SBR, while in SUS the Benin haplotype predominates. The HBB haplotypes were firstly believed to indicate five distinct HbS mutation events, but a recent report favours the hypothesis of a single origin of the HbS allele in Africa approximately 7,300 years ago^19^, while another study, taking population structure, demography, overdominance and balanced selection into account, estimated the origin of HbS mutation to have taken place approximately 22,000 years ago in the ancestors of African agriculturalists^20^. By evaluating 20 haplotypes containing the HbS in the 1,000 Genomes Project and in Qatar subjects, Shriner and Rotimi identified three clusters resulting from two split events. The first occurred on the ancestral haplotype and accounts for the CAR, Cameroon and Indian-Arab haplotypes, while the second gave rise to two clusters, one accounting for the Senegal and the other accounting for the Benin haplotypes^19^. The authors proposed that HbS had a single origin in the Sahara or in West-Central Africa, and a population diverged in present-day Cameroon, carrying the first cluster east and south as part of the Bantu expansion, while a separate migration wave headed north and west to present-day Senegal and the Gambia, giving rise to the Senegal and Benin haplotypes^19^.

Moreover, we propose that the divergence on the chromosome 11 is due to a recent selection event in the SBR population. We tested the genotyping rate for this range and found no missing data for either population, and the proportions of HBB haplotypes are in agreement with those reported for both cohorts^21^. Selection-suggestive signals seem to agree on a 100 kb region, as evaluated by LD, haplotype pattern, F_ST_, iHS and XP-EHH (Figs 2 and 3), ranging from chromosome 11:5.4 Mb to 5.5 Mb. This range comprises the LCR, a regulatory element well known for modulating the expression of gamma-globin. Low iHS values in the Brazilian patients overlap with a region also known to harbour an olfactory receptor cluster that has been associated with HbF production^22^. Additionally, we detected markers significantly associated with HbF in a group of 68 Brazilian patients after correcting for age, sex and hydroxyurea treatment (Fig. 3d). This finding supports the hypothesis of a selection event driven by an HbF modulating variant. Our data seem to be consistent with those of the study by Creary *et al*.^10^, who reported an association between European ancestry and the proportion of erythrocytes containing HbF. Another study, from Leonardo *et al*. evaluated variants in 244 sickle cell patients and found rs9399137 in the HMIP-2 locus, a relatively common European polymorphism, significantly associated with HbF levels^23^. The relationship between European background and clinical outcome is, therefore, far from established.

An alternative explanation for the local ancestry results is that the signals are a by-product of malaria related selection acting on the sickle cell allele. A hypothetical higher incidence of malaria in Brazil compared to the United States throughout history (malaria was controlled for most of the United States of America territory from the beginning of the twentieth century on^24^) could influence LD patterns and generate the aforementioned results. We, then, tested LD between the rs334 mutation region and the region under selection and found that they form independent blocks, not exceptionally linked at any marker (Supplementary Fig. S3). Moreover, F_ST_ values between affected and unaffected Brazilians are as high as 0.76 in this region, implying that the putative selection event acts strictly on SCA subjects and is related to the disease and not to the general population.

This study was limited by the relatively small sample sizes in SCA cohorts derived from just three sampling localities. These limitations make it difficult to extrapolate the results to larger and more broadly distributed sickle cell individuals from the two countries evaluated and also amplify statistical noise. Although assessed in regard of IBD, individuals might still have cryptic structure/consanguinity that would especially affect the LD patterns observed for Brazilian patients. Differences in gene flow, HbS allele frequency and HBB haplotype composition between sampled subjects from Recife and Campinas may have introduced variance not accounted in the analysis. Although genotyping rate is near 100% for markers included in ancestral analysis (see Methods), technical constraints may apply, as the inference of haplotype phase by population data is known to have greater switch error rates. Lo *et al*. evaluated major phasing algorithms and their accuracy through variation of panels and sample sizes, as well as by comparing trio and populational phasing and found SHAPEIT to yield a 3.52–6.51% switch error rate in small unrelated datasets (N from 15 to 32)^25^, while Choi *et al*. found SHAPEIT switch error to be 2.8% when phasing 85 unrelated individuals from European origin^26^. We would thus expect our data to fall into the range of approximately 3–6% switch error rate. The local ancestry inference might also be affected by the use of East Asian reference data as proxy of ancestral Americans, since it might inflate the estimates of haplotype contribution of that particular population^27^.

Here, we quantified divergence between two small cohorts and found this to be a promising way to highlight regions of high divergence that might be of functional importance or to uncover candidate loci based on selection signals. The haplotype structure has important implications on the cis-acting factors leading to variation on HbF production. More generally, these findings underline that the five RFLP-haplotype classifications proposed do not account for population-specific demographic factors and, while still useful, should be analysed carefully.

Genetic studies struggle to deal with admixture and other complex population demographic characteristics in face of association to phenotypic traits. Admixture mapping, a tool to perform this task, has been recently developed and relies on regions of different allele frequency driven by contrasting ancestries. It has been suggested that admixture mapping may only be applicable when ancestral populations differ in the phenotype of interest^8^, and this seems to be the case for SCA patients with regard to HbF production. Admixed mapping, nonetheless, has been applied when the ancestral populations are European and African^10,28–31^. It is still a matter of debate whether HbF levels are influenced by European ancestry^8^, whereas different ancestries inside the African continent have already been proven to be diverse regarding gamma-globin expression. Moreover, it is still unclear how different levels of admixture will translate to HbF production and other phenotypic traits. Sickle cell disease ancestry studies could lead to novel loci associated with phenotypic variability. Here we demonstrate that SCA samples from different locations may largely vary on the genomic and local ancestry on chromosome 11. Further studies in larger cohorts, sampled from different locations are welcomed to better describe the variation in ancestral background on genomic and HBB cluster levels. Also, more detailed migration history data and the advancement in fine structure inference methods will broaden our understanding of how patterns of gene flow, admixing, selection and linkage disequilibrium act on shaping genomic regions that impact important phenotypic human traits.

In conclusion, we found the two different cohorts of SCA to differ in both genome-wide ancestral composition and locally to the causal locus region. Comparing admixed populations may be a strategy to reveal regions of local adaptation that would otherwise require a large association study to be unveiled.

## Material and Methods

### Ethics statement

The present research followed the principles of the Declaration of Helsinki; all patients were presented to the aims and details of the study and signed an informed consent. The institutional review board committee at CHOP and Ethics Committee at the Faculty of Medical Sciences at UNICAMP approved subjects’ enrolment, blood collections and study methods.

### Subjects

The complete dataset comprises a total of 1,994 individuals, 1,822 of which are part of the 1,000 Genomes repository (http://www.internationalgenome.org/data/) representing global populations (Supplementary Table S1). Brazilian SCA patients were recruited at HEMOPE (Recife, Pernambuco; n = 57) and HEMOCENTRO (Campinas, São Paulo; n = 34) haematological therapy centres, along with 51 unaffected Brazilians from HEMOCENTRO (n = 31) and from the project “Assessment of Copy Number Variation in Congenital Defects of Complex Inheritance”^32^, also collected in Campinas (n = 20). The American cohort data is composed of 30 SCA patients with data filtered out from the Epic Care Clinical System (Epic, Verona, WI), along with 60 auto-declared African Americans not affected by sickle cell diseases, all from CHOP, Pennsylvania. Differently from African-Americans, the Brazilian group of unaffected subjects (HbAA) was not selected regarding skin pigmentation or self-declared African background, since the SBR population is already known to be more heterogeneous in ancestral composition^8^.

### Genotyping

Genotyping of both American and Brazilian samples was carried out on the Affymetrix Genome-Wide Human SNP 6.0 array platform (Affymetrix Inc., CA, USA), according to manufacturer’s protocol. Genotype data was analysed along with reference populations from 1000 Genomes Project^33,34^. We selected 19 reference populations from the African, European, American and Asian continents, as shown on Supplementary Table S1.

### Quality control (QC)

Processing of raw genotype data and the basic quality control procedure was performed with the aid of the PLINK v1.9 software^35^. Each individual sample was checked for discordance in relation to the sex register, outlying missing genotype call rate (genotyping rate ≥ 0.90 were kept); we also evaluated relatedness in the collected samples by calculating genome-wide identity-by-descent (IBD), removing one sample from pairs of duplicates or pairs estimated to be second-degree relatives or closer (IBD < 0.1875 were kept, see Supplementary Fig. S5). Each population was evaluated for genotyping quality and markers consistency throughout the sample, removing markers with low minor allele frequency (<0.01); or demonstrating deviation from Hardy-Weinberg equilibrium (HWE), p-value < 10^−9^. We also composed a list of SNPs for which at least one Mendelian inconsistency was observed in populations that had information for trios, excluding such SNPs from further analyses. The final genotyping call rate was 0.9993 for genomic analysis and 1.0 and 0.9950 for chromosome 11 in SBR and SUS, respectively.

### Linkage disequilibrium (LD)

For performing PCA, we have controlled the data for regions of high LD through the extraction of local and long-range markers in LD (r^2^ < 0.5) as the first step. We also excluded regions of known extensive LD across the genome^36^. For displaying regions of LD we used Haploview v4.2^37^, SNPs with strong LD (D′ ≥0.8) were considered part of a haplotype block using confidence intervals as proposed by Gabriel and colleagues^38^.

### Genome-wide population structure

Genome-wide population structure and admixture were analysed by principal component analysis (PCA), using EIGENSOFT v7.2.1^39^; and an ancestry modelling approach implemented in ADMIXTURE v1.3.0^40^, while R software was used to generate graphical representation of the results. EIGENSOFT applied PCA, a non-parametric technique for reducing the multidimensionality to orthogonal eigenvectors that enclose the maximum variance to the genotypic data, in data from all 23 populations. Data is converted to a matrix representing individuals and their genotypes for 155,820 SNPs kept after QC and LD treatment. Eigenvectors representative of the largest amount of variance in data were then used to build the PCA plot. We also calculated the F-statistic among populations, by the Hudson’s F_ST_ method, also implemented in the EIGENSOFT package. ADMIXTURE software assigns individuals, on the basis of differences in allelic frequencies by maximum likelihood estimation, to ancestry clusters (K). We identified the optimal value of K (6) by the least error cross-validation method after testing K values ranging from 1 to 18.

### Local ancestry inference

We phased genotypes of the chromosome 11 on both Brazilian and American SCA patients using the SHAPEIT v2.r790 method^41^. We then analysed local ancestry in this chromosome using the software SABER+ v1.0, which implements a Markov-Hidden Markov Model for inferring locus-specific ancestry in admixed individuals^42^. We modelled SCA cohorts as a mixture of chromosomes from three ancestral populations with various global proportions of European, Native American and West African ancestries. Although real admixture histories are more complex than this, we simplified them for the sake of data tractability, since more convoluted admixing models are still poorly addressed by current algorithms^43^.

We considered admixed haplotypes as mosaics of segments derived from three of the HapMap phase 3 haplotype panels^34^: phased haplotypes from the CEU (117 haplotypes), CHB + JPT (169) and YRI (115) trio-phased panels, as proxy haplotype data from Europeans, Native American and African ancestors, respectively. We also applied a Weir & Cockerham’s makerwise F_ST_ estimation^44^ implemented in VCFLIB package (https://github.com/vcflib/vcflib). Haplotypes blocks images were generated in Haploview^37^ and VCFLIB.

### HBB haplotypes inference

For evaluating inference in the classical 5 HBB haplotypes we used phased haplotypes of SCA subjects to impute not-typed markers on chromosome 11, including 4 SNPs (rs3834466, rs28440105, rs10128556, and rs968857) that define these haplotypes as described in Shaikho *et al*.^45^. Imputation was performed by IMPUTE2 v2.3.2 method^46^.

### Integrated haplotype score (iHS)

The integrated haplotype Score (iHS) was proposed by Voight *et al*. as a method to describe events of recent selection^47^. iHS is the amount of extended haplotype homozygosity (EHH) at a given marker along the ancestral allele relative to the derived allele empirically standardized to mean of 0 and variance of 1. Values lower than −2 (for ancestral allele) or higher than 2 (for derived allele) are regarded as signals of recent positive selection. A stretch of extended homozygosity for haplotypes on a high frequency allele relative to the other is a signature of a sweep resulting from positive selection. We computed iHS values for SCA from Brazil and the US using the VCFLIB package. By linearly interpolating between SNPs, EHH was integrated with respect to genetic distance for markers that reached EHH of 0.05 in both directions from the core SNP, otherwise, that SNP was skipped. Normalization is then performed to account for regional differences in allele frequencies.

### Cross-population extended homozygosity (XP-EHH)

Cross Population Extended Haplotype Homozygosity detects sweeps resulting from selected alleles that have trend towards fixation in one population but not the other^48^. We used the *selscan* v1.1.0 software to perform XP-EHH calculation^49^.

### Association test

We selected the GRCh37 chr11:5.54–5.59 Mb region on account of the F_ST_ values shown in Fig. 3c. In this region, F_ST_ values are high between Brazilian and American cohorts but drop between Brazilian and Iberian populations. We modelled HbF as response variable on a linear regression, performing a single test for each of the 31 SNPs as predictor variables, along with age, sex and hydroxyurea treatment as covariates. All variants had MAF >0.05 and association with HbF was tested using a standard linear regression of phenotype on allele dosage implemented in PLINK v1.9^35^. The gvlma R package^50^ was used to test fitness of data to the linear regression assumptions. We provide plots showing normality of residuals for significant SNPs in Supplementary Fig. S6. A significance level of 0.05 was adopted and Bonferroni adjustment applied for correcting for multiple testing, We generated a local association plot using the LocusZoom v1.4^51^ tool (see Supplementary Fig. S4).

## Supplementary information

Supplementary Information

## Data Availability

The datasets generated and/or analysed during the current study are available from the corresponding author on reasonable request.
