# Supplementary material for: Genetic comparison of sickle cell anaemia cohorts from Brazil and the United States reveals high levels of divergence
Source: Sci Rep. 2019 Jul 26;9:10896. doi: 10.1038/s41598-019-47313-2 (PMC6659681; doi:10.1038/s41598-019-47313-2)
Supplement: Supplementary file 1 — Supplementary Information [file 41598_2019_47313_MOESM1_ESM.pdf]

Genetic comparison of sickle cell anaemia cohorts from Brazil and the United States reveals high levels of divergence

Pedro R. S. Cruz[1], Galina Ananina [1], Vera Lucia Gil-da-Silva-Lopes[2], Milena Simioni[2], Farid Menaa[1], Marcos A. C. Bezerra[3], Igor F. Domingos[3], Aderson S. Araújo[4], Renata Pellegrino[5], Hakon Hakonarson[5], Fernando F. Costa[6], Mônica Barbosa de Melo[1]*

*Correspondence and requests for materials should be addressed to melomb@uol.com.br.

Table S1. Geographical and ethnic origin for each population under analysis.

Populations' labels	Description
CEU	Utah residents with Northern and Western European ancestry from the CEPH collection
FIN	Finnish in Finland
CHB	Han Chinese in Beijing, China
JPT	Japanese in Tokyo, Japan
TSI	Tuscans in Italy
ASW	Americans of African Ancestry in SW USA
LWK	Luhya in Webuye, Kenya
YRI	Yoruba in Ibadan, Nigeria
CLM	Colombians from Medellin, Colombia
MXL	Mexican Ancestry from Los Angeles USA
AAM	African American from Philadelphia; USA
SUS	Sickle cell anemia patients from Philadelphia USA
BRZ	Brazilians from São Paulo; Brazil
SBR	Sickle cell anemia patients from Pernambuco and São Paulo; Brazil
GBR	British in England and Scotland
IBS	Iberian Population in Spain
GWD	Gambian in Western Divisions in the Gambia
MSL	Mende in Sierra Leone
ESN	Esan in Nigeria
ACB	African Caribbeans in Barbados
NAM	Aymara/Quechua from Bolivia and Quechua from Peru [83].
PUR	Puerto Ricans from Puerto Rico
PEL	Peruvians from Lima, Peru

Table S2. **Hudson's F_{ST} statistics estimated between each pair of populations.** This analysis was conducted on 155,820 SNPs.

	NAM	AAM	SUS	BRZ	SBR	GBR	FIN	PUR	CLM	IBS	PEL	ACB	GWD	ESN	MSL	CEU	YRI	CHB	JPT	LWK	ASW	MXL
AAM	0.169																					
SUS	0.162	0																				
BRZ	0.116	0.026	0.022																			
SBR	0.115	0.021	0.017	0.001																		
GBR	0.139	0.094	0.086	0.025	0.033																	
FIN	0.132	0.095	0.088	0.029	0.036	0.007																
PUR	0.104	0.061	0.055	0.009	0.013	0.011	0.015															
CLM	0.08	0.071	0.065	0.015	0.019	0.016	0.019	0.006														
IBS	0.141	0.089	0.082	0.022	0.03	0.002	0.01	0.009	0.015													
PEL	0.012	0.122	0.116	0.068	0.069	0.086	0.081	0.057	0.039	0.087												
ACB	0.177	0.001	0.001	0.033	0.027	0.104	0.106	0.07	0.081	0.1	0.131											
GWD	0.197	0.007	0.009	0.05	0.043	0.129	0.13	0.091	0.102	0.124	0.151	0.006										
ESN	0.199	0.006	0.007	0.051	0.044	0.133	0.134	0.095	0.106	0.128	0.154	0.004	0.008									
MSL	0.199	0.006	0.008	0.052	0.044	0.133	0.134	0.095	0.105	0.128	0.154	0.005	0.004	0.005								
CEU	0.139	0.093	0.086	0.025	0.032	0	0.006	0.011	0.016	0.002	0.085	0.104	0.128	0.133	0.132							
YRI	0.198	0.005	0.007	0.05	0.043	0.132	0.133	0.094	0.104	0.127	0.153	0.003	0.006	0.001	0.004	0.131						
CHB	0.109	0.126	0.12	0.085	0.085	0.105	0.098	0.082	0.074	0.105	0.08	0.134	0.152	0.155	0.155	0.105	0.154					
JPT	0.108	0.128	0.122	0.086	0.087	0.107	0.099	0.084	0.076	0.107	0.081	0.136	0.154	0.157	0.156	0.107	0.156	0.007				
LWK	0.191	0.007	0.008	0.045	0.038	0.123	0.124	0.086	0.096	0.117	0.145	0.007	0.011	0.008	0.009	0.122	0.007	0.146	0.148			
ASW	0.155	0.001	0	0.019	0.015	0.081	0.083	0.05	0.059	0.077	0.109	0.003	0.011	0.01	0.01	0.08	0.009	0.115	0.117	0.01		
MXL	0.046	0.087	0.08	0.03	0.032	0.037	0.036	0.019	0.009	0.037	0.017	0.096	0.117	0.121	0.121	0.036	0.12	0.066	0.067	0.111	0.074	
TSI	0.142	0.09	0.083	0.023	0.03	0.004	0.012	0.01	0.016	0.002	0.087	0.101	0.125	0.129	0.129	0.004	0.128	0.105	0.107	0.118	0.078	0.037

Table S3. SNPs tested for association to HbF in the Brazilian cohort. SNPs in bold are significantly associated to HbF after Bonferroni's correction.

SNP	hg19 pos.	N	Beta	p-value	Corrected p-value
rs12418972	5540466	68	4.179	0.01152	0.31332
rs12286144	5543019	68	0.9452	0.8548	1
**rs1433567**	**5544512**	**68**	**5.718**	**0.0003109**	**0.0096379**
rs11037947	5544589	68	4.179	0.01152	0.31332
**rs2010794**	**5545822**	**68**	**5.044**	**0.001533**	**0.04599**
rs10160557	5546722	68	3.318	0.04419	0.97218
rs10160561	5546809	68	3.779	0.02166	0.51984
rs7932885	5547530	68	0.4407	0.7244	1
rs4910815	5547957	68	3.446	0.05707	0.97218
rs10838263	5548214	68	0.03746	0.4817	1
rs10838273	5552167	68	0.03746	0.4817	1
rs167602	5561595	68	-2.998	0.1115	1
rs1566275	5566365	68	5.864	0.2785	1
rs12274861	5567019	68	4.652	0.005539	0.160631
rs431702	5569452	68	3.171	0.08962	1
rs368019	5569528	68	3.495	0.03951	0.90873
rs10500651	5573580	68	2.164	0.9075	1
rs2647602	5573701	68	3.546	0.04627	0.97218
rs431117	5574280	68	3.546	0.04627	0.97218
rs446099	5574493	68	3.546	0.04627	0.97218
rs430197	5574641	68	3.546	0.04627	0.97218
rs407487	5575382	68	3.546	0.04627	0.97218
rs1541936	5578584	68	-4.597	0.0126	0.315
rs16932946	5585716	68	4.231	0.01119	0.31332
rs10838341	5585744	68	2.924	0.2019	1
rs10769072	5585884	68	-1.011	0.107	1
rs10742719	5586252	68	5.91	0.04811	0.97218
rs10742722	5586372	68	5.91	0.04811	0.97218
rs7124406	5598243	68	1.07	0.9311	1
rs7128534	5599182	68	0.02612	0.6839	1
rs11038174	5599901	68	-3.671	0.3143	1

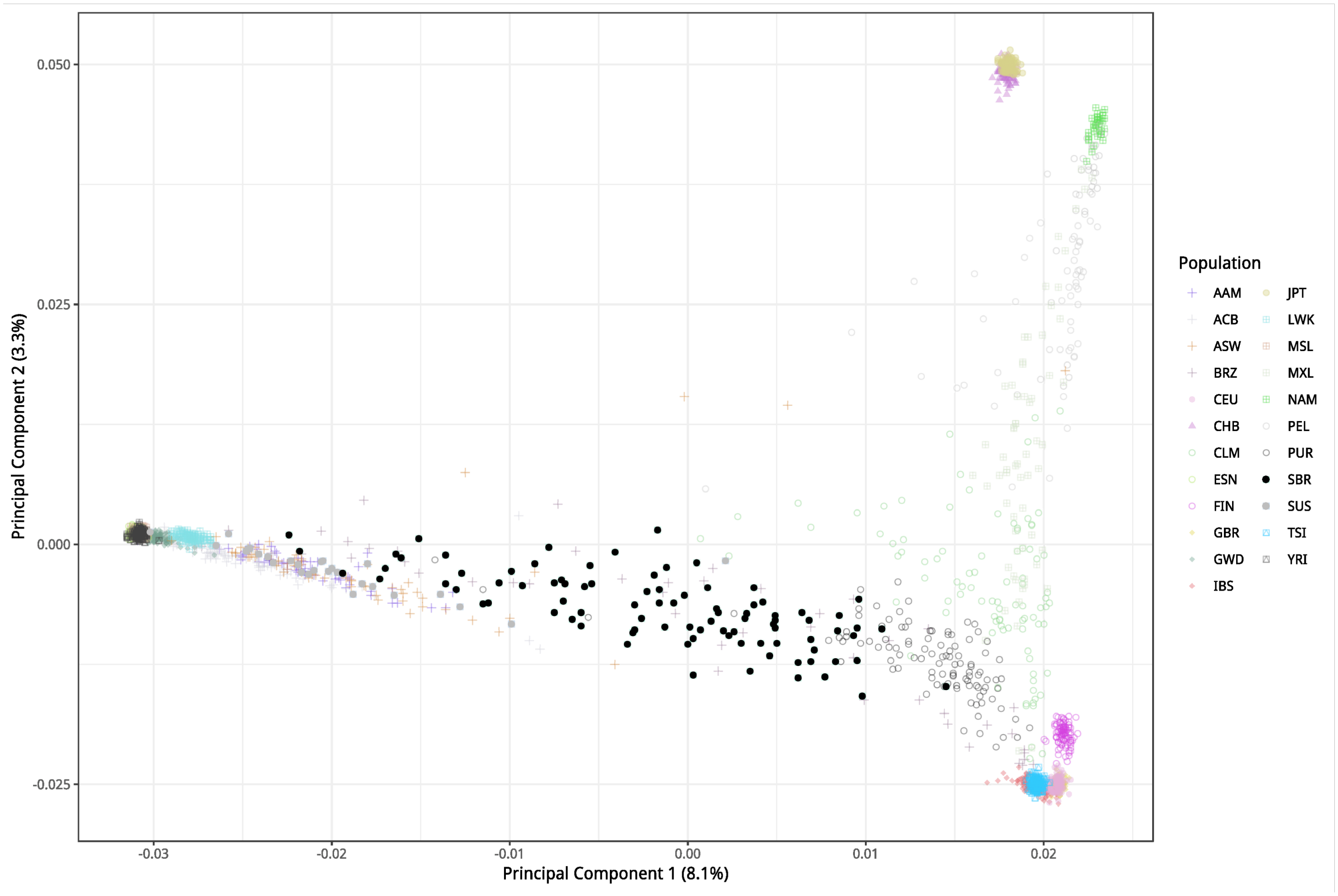

Figure S1. Plot of the Principal Component Analysis (PCA). The PCA was performed on 155,820 markers. Sickle cell cohorts are highlighted in solid colours: gray – American sickle cell patients, black – Brazilian sickle cell patients. Variability accounted by each PC is depicted in parenthesis.

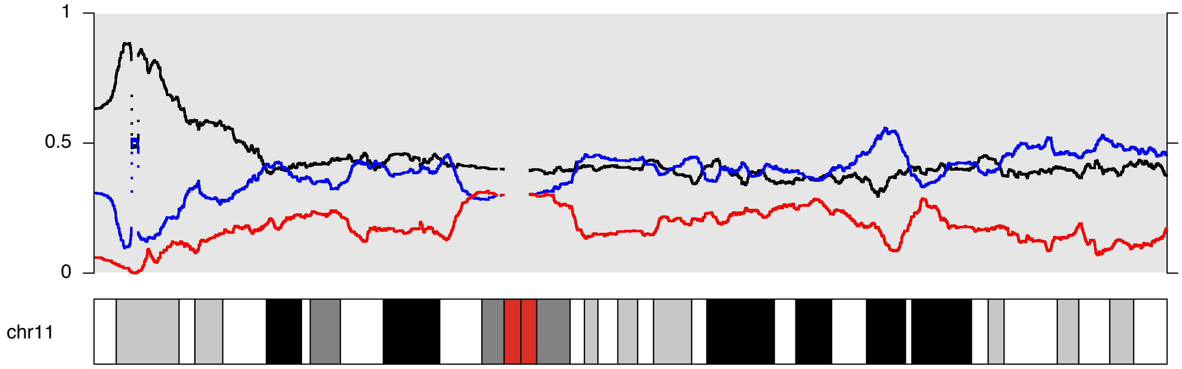

Figure S2. Estimated continental ancestry proportion for sickle cell anaemia patients from Brazil along chromosome 11. Dark line: African ancestry, blue line: European ancestry and red line: Native American ancestry.

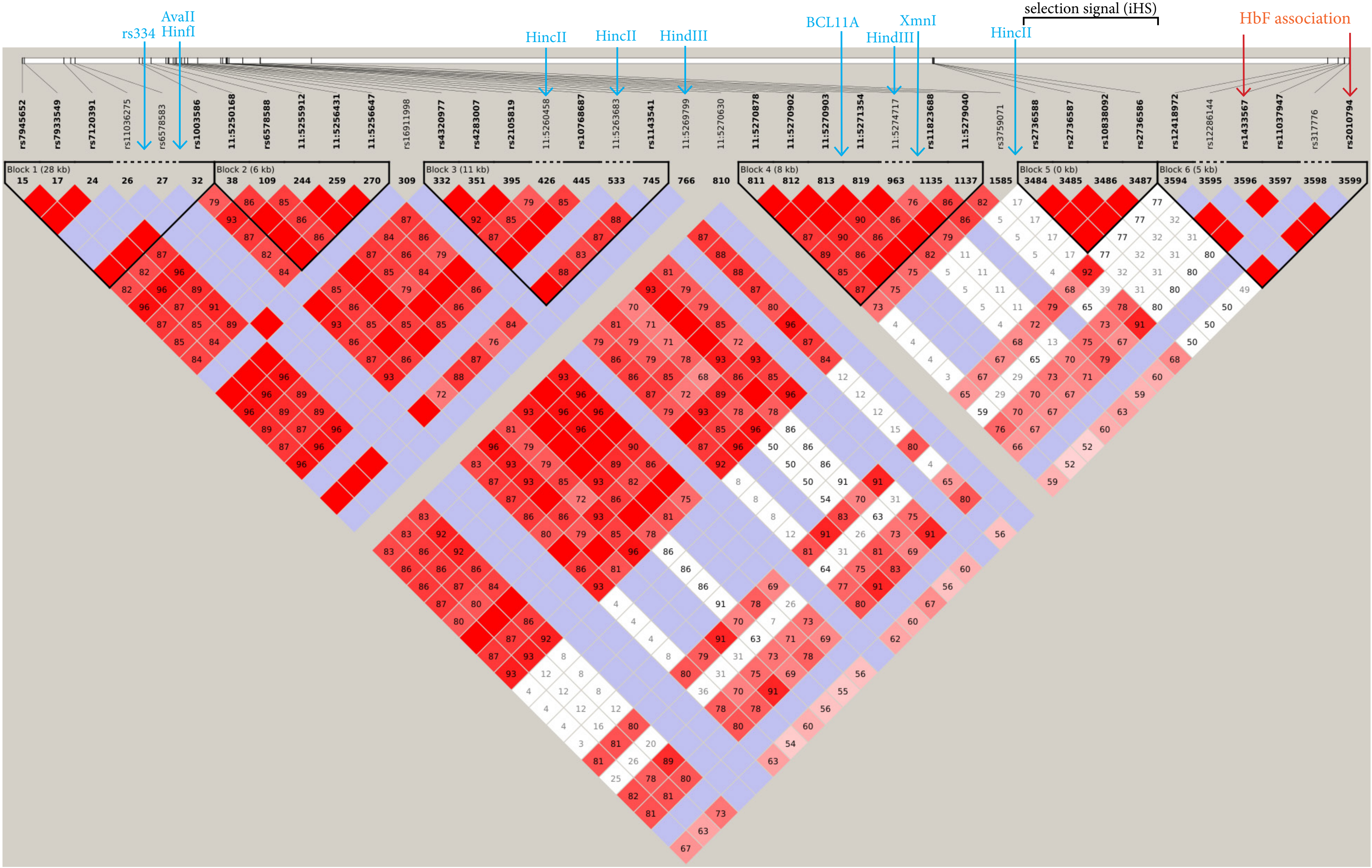

Figure S3. Linkage Disequilibrium blocks in SCA patients from Brazil (SBR) on the chr11:5.220-5.545Mb range. Relative (long arrows) or actual (short arrows) locations of HbS mutation (rs334), restriction enzymes sites used on HBB haplotype classification (HinfI, AvaII, HincII, HindIII and XmnI), BCL11A binding site and SNPs in association with HbF (red). SNPs under the bracket suggest positive selection in the region. Only informative markers (tag SNPs) were kept to represent LD blocks, markers in the 'chr:pos' format were imputed by IMPUTE2.

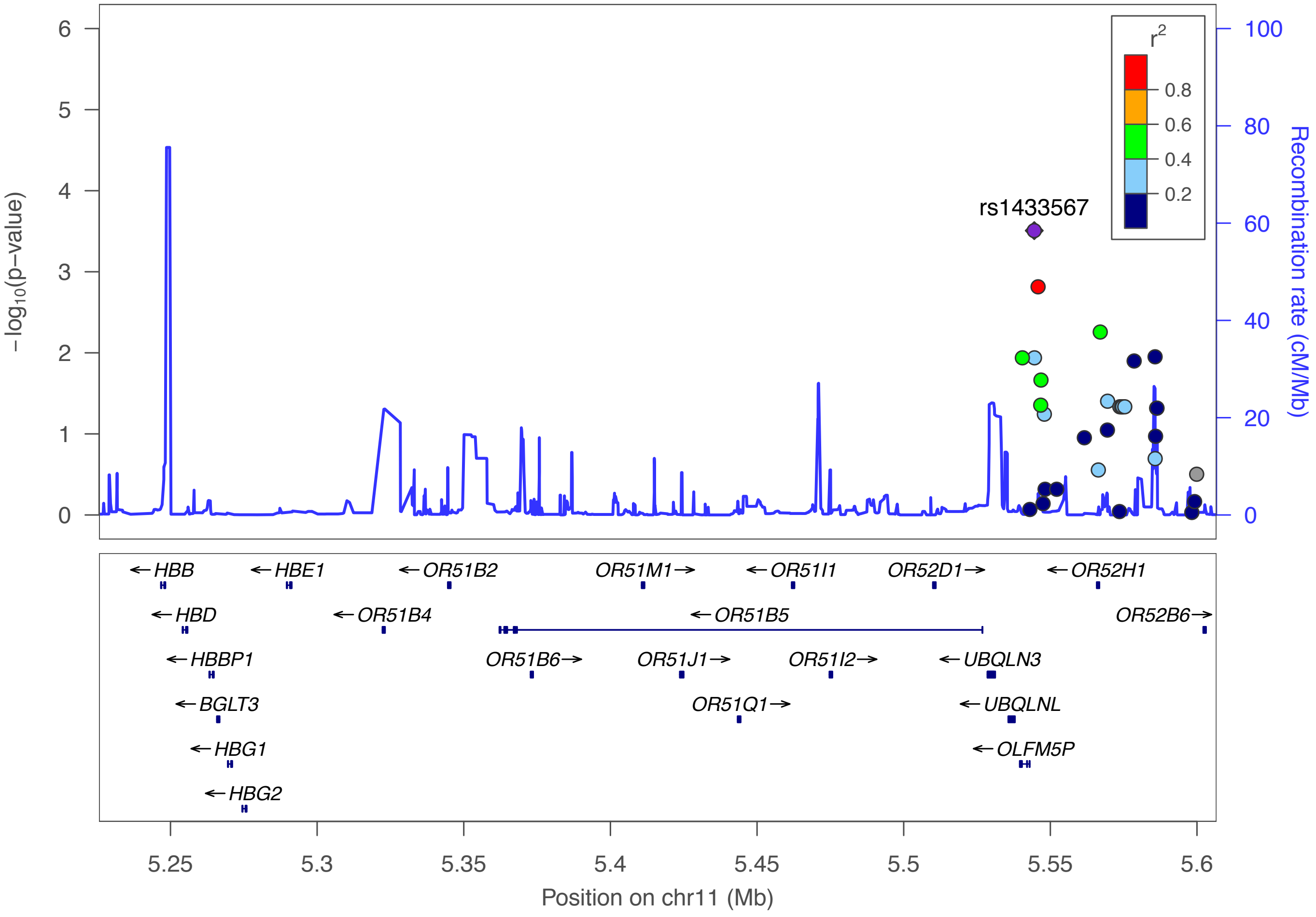

Figure S4. Local association for markers on coordinates chr11:5.2 to 5.6 Mb (31 SNPs). Tested SNPs along with genomic context (below), recombination rate (blue line) and LD (r^2) information. Nominal p-values are depicted by left vertical axis, while recombination rate values are on the right.

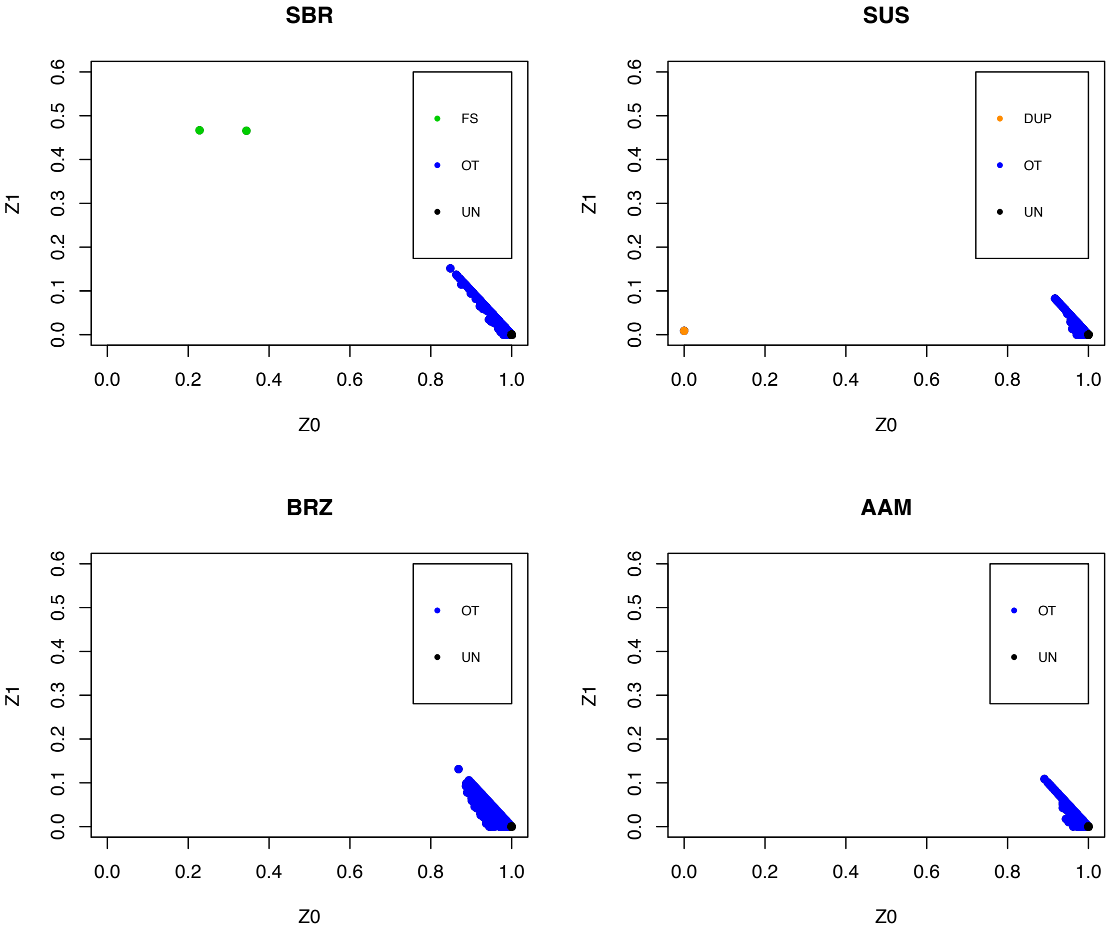

Figure S5. Probabilities that pairs of individuals share zero (Z=0) or one (Z=1) allele identical-by-descent (IBD) on the four sampled populations. Pairs are represented as dots and colours represent inferred duplication/relatedness. DUP – duplicated (or monozygotic twins); FS – full siblings; OT – other related; UN – unrelated.

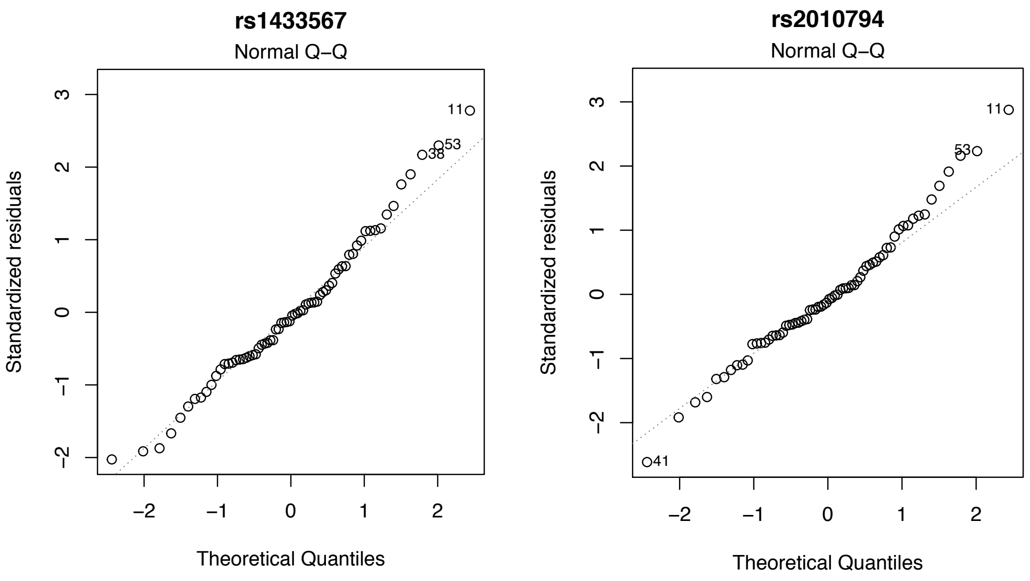

Figure S6. Comparison of the theoretical to the empirical quantiles of the residuals for SNPs rs1433567 and rs2010794. Residuals of the performed linear regressions showing approximate normality; numbered circles represent outliers.
